# Malaysian Cobra Venom: A Potential Source of Anti-Cancer Therapeutic Agents

**DOI:** 10.3390/toxins11020075

**Published:** 2019-02-01

**Authors:** Syafiq Asnawi Zainal Abidin, Yee Qian Lee, Iekhsan Othman, Rakesh Naidu

**Affiliations:** 1 Liquid Chromatography Mass Spectrometry (LCMS) Platform, Monash University Malaysia, Jalan Lagoon Selatan, Bandar Sunway 47500, Selangor Darul Ehsan, Malaysia; syafiq.asnawi@monash.edu (S.A.Z.A.); iekhsan.othman@monash.edu (I.O.); 2 Jeffrey Cheah School of Medicine and Health Sciences, Monash University Malaysia, Jalan Lagoon Selatan, Bandar Sunway 47500, Selangor Darul Ehsan, Malaysia; Yee.Lee@monash.edu

**Keywords:** snake venom, Malaysian cobras, *N. kaouthia*, *N. sumatrana*, *O. hannah*, anticancer

## Abstract

Cancer is a deadly disease and there is an urgent need for the development of effective and safe therapeutic agents to treat it. Snake venom is a complex mixture of bioactive proteins that represents an attractive source of novel and naturally-derived anticancer agents. Malaysia is one of the world’s most biodiverse countries and is home to various venomous snake species, including cobras. *Naja kaouthia*, *Naja sumatrana*, and *Ophiophagus hannah* are three of the most common cobra species in Malaysia and are of medical importance. Over the past decades, snake venom has been identified as a potential source of therapeutic agents, including anti-cancer agents. This present review highlights the potential anticancer activity of the venom and purified venom protein of *N. kaouthia, N. sumatrana*, and *O. hannah*. In conclusion, this review highlights the important role of the venom from Malaysian cobras as an important resource that researchers can exploit to further investigate its potential in cancer treatment.

## 1. Introduction

Cancer is a major health problem that affects people all over the world. Globally, 25% of human mortality is due to cancer. In the United States, cancer was reported as the second leading cause of death in 2015. Approximately 1,735,350 new cancer cases and 609,640 deaths due to cancer were expected in 2018 [1]. In Malaysia, cancer remains one of the leading causes of death, and a total of 64,725 deaths were reported from 2007 to 2011 [2]. Cancer is a group of diseases that arises from the uncontrollable proliferation of malignant cells. It is a multigenic and multistage disease due to multifactorial etiology [3]. Briefly, high levels of exposure to carcinogens such as radiation, tobacco, and oncogenic viruses increase the risk of DNA damage in the cells. The DNA-repair mechanism will be initiated at this stage. However, when the damage is too extensive, the repair of lesions fails. This gives rise to changes in the expression of genes (such as tumor-suppressor genes) in the cells, which further alter the signalling pathways resulting in unrestricted cell growth [4].

Even though the incidence of cancer is increasing globally, the mortality rate of cancer is reported to be in decline for the past 20 years [5]. This may be due to the advancement of therapeutic regimes over the past few decades. Various therapeutic options such as surgery, chemotherapy, radiotherapy, and immunotherapy are employed in treating localized cancer. Surgery in combination with chemotherapy is still the main treatment option. Unfortunately, owing to its cytotoxic activity via the inhibition of nucleic acid synthesis, chemotherapy often results in the death of fast growing cells such as white blood cells, hair follicles, and cells lining the gastrointestinal tract in addition to cancer cells [6]. Therefore, patients often suffer from side effects such as nausea and hair loss. A weakened immune system due to the reduction of white blood cell levels during chemotherapy increases patients’ susceptibility to infection. The development of drug resistance during chemotherapy further complicates the treatment of cancer. Hence, there is an urgent need for effective cancer therapeutics with lesser side effects.

Malaysia, as the 12th most biodiverse country in the world, is the home to approximately 170,000 species of flora and fauna. Since ancient times, natural resources have been exploited to treat diseases and improve human health. For instance, the use of *Orthosiphon aristatus* (Misai Kuching) as a natural remedy against diabetes is common among indigenous communities in Malaysia [7,8]. Poisonous animals also play a vital role in the discovery of novel therapeutic candidates. For example, bee venom therapy is used to relieve pain symptoms and treat diseases such as rheumatoid arthritis [9,10] and other neurological diseases [11,12]. Venom from the Indian black scorpion was found to induce DNA fragmentation and reduce the proliferation of human leukemic cells [13]. Additionally, a novel peptide named Gonearrestide from scorpion venom showed the inhibition of primary colon cancer cells and solid tumor growth [14]. Commercialized drugs such as Captopril^®^ and Enalpril^®^ are two successful antihypertensive drugs developed based on bradykinin peptides derived from the venom of the snake *Bothrops jararaca* [15,16]. Ziconotide is another FDA-approved analgesic medication derived from ω-conotoxin that was found in the venom of *Conus magus*, a marine snail [17]. By reviewing the pharmaceutical potential of animal venoms, we conclude that the complex mixture of proteins may have the potential to be an important source of therapeutic agents. 

Snake venom has been associated with various therapeutic applications—as a thrombolytic agent in cardiovascular disorders [18], anti-microbial activities [19], as an anti-viral agent [20] and in antiparasitic, and antifungal activities [21,22]. Undeniably, the anticancer activities of snake venom represent one of its most attractive therapeutic features and they have been actively researched and reviewed over the past decade [23,24,25]. The venom from Malaysian common cobras has been characterized, and proteins with anticancer potential have been described. However, while there are numerous reviews focusing on the anticancer activities of snake venom in general, none have focused on the Malaysian common cobra species, i.e., *Naja kaouthia*, *Naja sumatrana*, and *Ophiophagus hannah*. The abundance of these cobra species provides valuable access for researchers to further investigate the venom activity. Therefore, the present review highlights the anticancer activity of the venom components of Malaysian cobra species.

## 2. Malaysian Common Cobras

Malaysian venomous snake species can be divided into two families, Viperidae and Elapidae [26,27]. Viperidae can be further divided into three families, Azemiopinae (Fea’s viper), Crotalinae (pit vipers), and Viperinae (true vipers). Malaysian vipers belong to the subfamily Crotalinae, which can be distinguished by the loreal pit on either side of the eyes [26]. Additional characteristics of pit vipers include hollow and retractile fangs on a moveable maxillary bone; a stocky, keel-scaled body with elliptical pupils; and that they are ovoviparous [26]. Elapidae is represented by cobras, kraits, and coral snakes that produce neurotoxic venom. It is characterized as a family of snakes with short and sharp fangs located anteriorly on the maxillary bone, with smooth-scaled body with rounded pupils, and that are oviparous [26]. Three cobra species, namely *Naja kaouthia*, *Naja sumatrana* and *Ophiophagus hannah* are the most common cobras in Malaysia. *N. kaouthia* or monocled cobra was formerly known as *Naja naja siamensis*, a subspecies of the Indian cobra (*Naja naja*) [26]. *N. sumatrana* is a spitting cobra and it is the most common Elapid of the ten species in the family. Both *N. kaouthia* and *N. sumatrana* can inhabit a wide range of environments, ranging from natural to anthropogenic landscapes. Members of the *Naja* genus are well known to be aggressive and envenomation is common for both species as humans infringe on their niche during the progress of urbanization. The third Malaysian cobra species is *O. hannah* or king cobra. *Ophiophagus*, meaning snake eater in Greek, is a monotypic genus, where the king cobra is the only species in this genus. It is the longest venomous snake species and is a dreadful assailant that is famous for its agility. The fatality rate incurred by king cobra envenomation is relatively high, although bites are rarely reported [26]. Table 1 provides a summary of comparisons between the cobras. In spite of their toxicity, the venom of Malaysian cobras demonstrates a wide range of therapeutic potential through antibacterial [28,29], anticancer [30,31,32,33], anticonvulsant [34], and antithrombotic [35] activities.

## 3. Proteomic Composition of the Venom from *N. kaouthia*, *N. sumatrana*, and *O. hannah*

Snake venom is a natural resource that can be readily obtained, especially from Malaysian *N. kaouthia*, *N. sumatrana*, and *O. hannah*. The evolutionary arms race has driven the diversification of toxins in snake venom. It is a complex mixture comprising: 1) proteins such as phospholipase A_2_ (PLA_2_), L-amino acid oxidase (LAAO), acetylcholinesterase, and protease; 2) peptides such as disintegrins; 3) low-molecular-weight organic compounds such as carbohydrates and histamines; and 4) inorganic ions such as magnesium, cobalt, iron, and potassium [40]. The cocktail of proteins in snake venom aids the snakes in capturing and digesting their prey. These proteins can be categorized as cytotoxins, hemotoxins, neurotoxins, and cardiotoxins [4]. However, the composition of snake venoms may have inter- and intraspecies variation, depending on habitat, diet, gender, and ontogenetic development [4,41]. 

The advancement of mass spectrometry techniques has allowed for the proteomic characterization of the venom from *N. kouthia*, *N. sumatrana*, and *O. hannah*. A combination of transcriptomic and proteomic analyses of *N. kaouthia* venom has identified proteins such as the three-finger toxin (3FTx), phospholipase A_2_ (PLA_2_), ohanin, cysteine-rich venom protein (CRVP), snake venom metalloproteinase (SVMP), venom nerve-growth factor (vNGF), cobra venom factor (CVF), cardiotoxin, cytotoxin, and neurotoxin [36,37]. The proteomic characterization of *N. sumatrana* venom identified proteins including PLA_2_, neurotoxins, cardiotoxin, cytotoxin, 3FTx, CVF, SVMP, CRVP, natriuretic peptide, aminopeptidase, thaicobrin, complement-depleting factor, kaouthin-1, vNGF, and cobra serum albumin [38]. Similar proteins, such as 3FTx, SVMP, PLA_2_, and LAAO, were also identified from the venom of *O. hannah* in addition to acetylcholinesterase (AChE), phospholipase B (PLB), 5’-nucleotidase (5’NUC), neprilysins, and cystatins [31,39].

**Figure 1:**
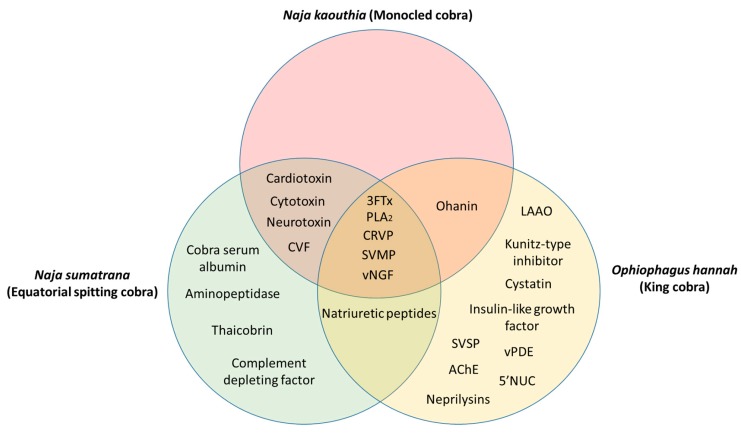
Common and unique proteins identified from the venom of *Naja kaouthia, Naja sumatrana*, and *Ophiophagus hannah.* Abbreviations: 3FTx—three-finger toxin, PLA_2_—phospholipase A_2_, CRVP—cysteine-rich venom protein, SVMP—snake venom metalloproteinase, vNGF—venom nerve-growth factor, CVF—cobra venom factor, LAAO—L-amino acid oxidase, vPDE—venom phosphodiesterase, SVSP—snake venom serine protease, AChE—acetylcholinesterase, 5’NUC—5’-nucleotidase.

The common and unique venom proteins from *N. kaouthia*, *N. sumatrana*, and *O. hannah* are summarized in Figure 1. Five proteins were found to be common to all three cobra species, namely, 3FTx, PLA_2_, CRVP, SVMP, and vNGF. Between *N. kaouthia* and *N. sumatrana*, four shared proteins were identified, including cardiotoxin, cytotoxin, neurotoxin, and CVF. Ohanin was found in both *N. kaouthia* and *O. hannah* and natriuretic peptides were identified in both *N. sumatrana* and *O. hannah*. Cobra serum albumin, aminopeptidase, thaicobrin, and complement-depleting factor were unique in *N. sumatrana* venom. Nine proteins in *O. hannah* venom were identified to be unique when compared with *N. kaouthia* and *N. sumatrana*, such as, LAAO, Kunitz-type inhibitor, cystatin, insulin-like growth factor, venom phosphodiesterase (vPDE), 5’NUC, snake venom serine protease (SVSP), AChE, and neprilysins.

## 4. Potential AntiCancer Activity of Malaysian Cobra Venom

The idea of utilizing snake venom as an important source of therapeutic agents and focusing on its anticancer properties has been extensively reviewed [23,24,42]. The investigation of snake venom’s effects on cancers can be traced back as early as the 1930s [43,44]. Since then, various snake venom proteins—most notably, LAAO, PLA_2_, SVMP/disintegrins, and snake venom C-type lectins (SNACLEC)—have been isolated and characterized for their activity as potential anticancer agents. The large amount of venom that can be obtained from the Malaysian common cobras renders them valuable for further investigation into potential therapeutic uses, especially as anticancer agents. The anticancer activity of the venom from the cobras is summarized in Table 2.

### 4.1. Ophiophagus Hannah

In a recent study by Kerkkamp et.al [45], the cytotoxic activity of crude *O. hannah* and *N. kaouthia* venom was demonstrated on the human pancreatic cancer cell line (PaTu 8988t) at an EC_50_ value of 1.39 ng/mL and 1.42 ng/mL, respectively. Selective cytotoxic activity was demonstrated by the crude venoms with EC_50_ values of approximately 20 ng/mL on the control cell lines (ZF4 cells; zebrafish cells) [45]. Furthermore, in-vitro migration and apoptosis assays demonstrated the ability of crude *O. hannah* venom to reduce cell migration activity and induce apoptosis, respectively [45]. Using an in-vivo zebrafish model, PaTu 8988t cells were injected post fertilization of the zebrafish to induce an angiogenic response. Treatment with *O. hannah* venom successfully inhibited the angiogenesis induction of the cancer cells [45].

LAAO is one of the major enzymatic protein components in *O. hannah* venom. The enzyme is categorized under flavoenzymes, which convert L-amino acid into alpha-keto acids with hydrogen peroxide (H_2_O_2_) as a byproduct [40]. The cytotoxic activity of LAAO from different snake species, including pit vipers and cobras, has been demonstrated in various human cancer cells [51,52,53]. The production of H_2_O_2_ was noted to be the main cause of cytotoxicity in several experiments [30,54,55,56]. The apoptosis-inducing activities of snake venom LAAO were also associated with the secondary production of H_2_O_2_ [57,58], but the specific mechanism remains unknown [40]. Interestingly, a study has demonstrated that LAAO was able to trigger the apoptotic mechanism even without the presence of H_2_O_2_ [59]. In 2014, Lee et.al [46] demonstrated the antiproliferative activity of LAAO purified from *O. hannah* (OH-LAAO) on human breast adenocarcinoma (MCF-7) and human lung adenocarcinoma (A549) with EC_50_ values of 0.04 µg/mL and 0.05 µg/mL, respectively. The cytotoxic activity was demonstrated to be selective on the cancer cells with greater potency when compared with doxorubicin, an established chemotherapeutic agent [33]. The induction of apoptosis partly contributed to the cytotoxic mechanism, as demonstrated by the increased level of caspase-3/7 and DNA fragmentation [33]. Similarly, OH-LAAO demonstrated cytotoxic activity against human prostate adenocarcinoma (PC-3) with an EC_50_ value of 0.05 µg/mL [32]. The in-vivo activity of OH-LAAO on nude mice implanted with PC-3 cells showed a significant reduction of tumor size with no obvious tissue damage in their vital organs [32]. These findings support an earlier study of OH-LAAO cytotoxicity on several cancer cell lines by Ahn et al. [30]. The investigators demonstrated the antiproliferative activity of OH-LAAO in murine melanoma cells (B16/F10) and human fibrosarcoma cells (HT1080) with approximately 74% inhibition at a concentration of 2 µg/mL. 

Fung and co-investigators [31] identified a total of 178 genes with significant alteration in MCF-7 cells treated with OH-LAAO. Amongst these were genes associated with the induction of apoptosis, such as, BMF (Bcl2 modifying factor), IGFBP3 (insulin-like growth-factor-binding protein 3), PLEKHF1 (Pleckstrin homology domain-containing, family F member 1), and PPARG (peroxisome proliferator-activated receptor gamma) [31]. Recent proteomic investigations by Fung et.al [46] further suggest that the use of OH-LAAO on MCF-7 induced 21 differentially expressed proteins with various biological functions including apoptosis, proteolysis, stress response, protein ubiquitination, and oxidoreduction. The authors concluded that the nonspecific oxidative modification of transcriptional factors caused by OH-LAAO is the key factor in the cell death and apoptosis induction. 

### 4.2. Naja Kaouthia

Feofanov et.al [48] demonstrated that cytotoxin (CT3) from *N. kaouthia* induced strong cytotoxic activity in human lung adenocarcinoma (A549) and human promyelotic leukemia cells (HL60) at an EC_50_ value of 2.6 µM and 0.18 µM, respectively. The authors further suggest that the cytotoxic effects of CT3 in HL60 were noted by their ability to bind strongly to the plasma membrane followed by internalization of the protein [48]. Furthermore, lysosomes were identified as the primary target of the cytotoxin that triggered the cytolytic action on the cells. [48]. Permeabilization of the plasma membrane was noted as a downstream event following lysosome rupture [48]. Interestingly, cytotoxins from other cobra species such as *Naja oxiana* and *Naja haje* demonstrated weak internalization of the protein in the plasma membrane compared to CT3. In a separate study investigating the anticancer activity of *N. kaouthia* crude venom by Debnath et.al [47], nonlethal doses of the crude venom inhibited the proliferation of various cancer cell lines such as Ehrlich-ascites cells (EAC), human lung lymphoblasts (U937), and human myelogenous leukemia cells (K562). Morphological changes associated with apoptosis such as membrane blebbing, chromatin condensation, and fragmentation were common features in cells treated with the *N. kaouthia* venom [47]. Furthermore, the solid-tumor growth of sarcomas using a Balb/c mice model was significantly reduced when treated with *N. kaouthia* crude venom [47]. 

In a follow-up study by Debnath et al. [49] on *N. kaouthia*, a lethal protein named cardiotoxic–cytotoxic protein was purified from the crude venom. The protein was identified through sequence homology to cytotoxins and cardiotoxins from the venom of other cobra species and demonstrated significant antiproliferative activity on human leukemic cells (U937 and K562) in a dose-dependent manner [49]. The leukemic cells treated with the cardiotoxic–cytotoxic protein demonstrated an increase of caspase-3/-9 activity and an increase of the proapoptotic Bax level, which suggest the induction of apoptosis [49]. Additionally, a novel protein, named Kaotree, has been identified and characterized from the venom of *N. kaouthia* with reports of selective cytotoxic activity on transformed mammary epithelial cells (HBL-100), mammary gland carcinoma (BT-20), breast cancer cells (ZR-75-1), and colon adenocarcinoma (HT-29) [50]. The anticancer activity of Kaotree was demonstrated to have an enhanced killing effect when combined with Atroporin, a snake venom protein derived from *Crotalus atrox* [50]. A patent was filed for the anticancer activity of Kaotree and Atroporin (US Patent No: 5565431) with the claim of a novel method for treating cancer patients.

### 4.3. Naja Sumatrana

While the anticancer activity of the crude venom of *O. hannah* and *N. kaouthia* has been well documented, to date, there is no available literature on *N. sumatrana* venom. However, proteomic characterization of *N. sumatrana* venom has identified major proteins with well-reported anticancer activity, such as PLA_2_, cardiotoxin, and neurotoxin [38]. The anticancer potential of snake venom PLA_2_ has been well reviewed [60] and the protein has been isolated from various snake species such as vipers [61,62], sea snakes [63], and cobras [64]. The cytotoxicity and anticancer activity of PLA_2_ has been demonstrated by MjTX-II, a PLA_2_ isolated from *Bothrops moojeni*, on treated EAC cancer cells, human breast carcinoma (SK-BR-3), and human T-cell leukemia (Jurkat) [65]. Ammodytoxin C, PLA_2_ purified from *Vipera ammodytes*, showed antitumoral activity against colon adenocarcinoma (Caco-2) [66]; and RVV-7, a cytotoxic PLA_2_ from *Daboia russellii*, displayed significant inhibition in B16F10 tumors in C57BL/6 mice [67]. In addition to its cytotoxic activity, snake venom PLA_2_ could also be employed as a model for drug development in humans. A study by Sales et al. [68] demonstrated similar interactions between PLA_2_ from *Bothrops* species and human-secreted PLA_2_ (HGIIA); which catalyzes the production of potent inflammatory molecules and is commonly associated with diseases [69]. Therefore, the anti-inflammatory activity of a novel drug can be studied using snake venom PLA_2_ for therapy models in humans. Cardiotoxin III (CTX-III) isolated from *Naja atra* venom has demonstrated anticancer activities in oral squamous cell carcinoma (Ca9-22) [70], human breast cancer cells (MDA-MB-231) [71], and human neuroblastoma cells (SK-N-SH) [72]. Cell death by CTX-III in all cell lines was attributed to the induction of apoptosis through a significant increase of caspase-3/-9 activity and cell cycle arrest. Alpha-cobrotoxin (α-CbT), a neurotoxin purified from *N. kaouthia* venom, prolonged the survival of animals in a non-small cell lung cancer (NSCLC) mouse model [73]. 

## 5. Future Directions and Conclusions

Cancer remains one of the most critical health burden worldwide, including in Malaysia. The disease presents a major challenge in the discovery and production of therapeutic agents that are effective, nontoxic, and cause fewer side effects. Anticancer agents from naturally derived sources—especially animal venom—have demonstrated their potential in cancer therapy. Venom-based protein/peptide therapeutics has been a subject of interest over the past few decades. Venomous animals such as scorpions and snakes were widely studied for their therapeutic values with some advantages and some challenges coming to light [24,74,75]. One of the major promises of a venom-based therapeutic agent is the specificity and the selectivity of its interaction with the target molecule. This is crucial to developing an anticancer agent with the ability to differentiate between normal and cancerous cells, with significant impact on cell proliferation, migration, and angiogenesis [76]. Moreover, some venom peptides, such as cytotoxins from *N. kaouthia* [47] are small and are able to penetrate cancer cells to trigger the cytotoxic effect. However, because of its toxic nature and our incomplete understanding of the anticancer mechanism of the venom proteins/peptides, the number of venom-based therapeutic agents currently in the market are very low [77].

Technological advancement in proteomics and genomics approaches, such as mass spectrometry and sequencing techniques, have allowed multiple proteins from venom to be isolated and characterized for activity. Proteins such as LAAO, PLA_2_, cytotoxin, and SVMP can be purified, characterized, and further investigated to determine the mechanisms by which they induce anticancer activity in vitro and in vivo. The majority of the studies mentioned in this review focused on the cytotoxicity of the venom/purified venom proteins on cancer cells, but further studies are needed to elucidate the potential anticancer mechanisms. Data obtained from the studies can serve as a template for further preclinical and clinical studies to demonstrate the safety and efficacy of these anticancer proteins. The delivery of the venom protein/peptide-based therapeutics represents another challenge in this field. However, continued advancements in the field of molecular biology, recombinant proteins, and drug delivery, such as nanoparticles, could possibly overcome the issues of bioavailability, pharmacokinetics, and efficacy of snake venom proteins as an anticancer drug. A study by Al-Sadoon et al. [77] demonstrated that the venom from *Walterinnesia aegyptia* combined with silica nanoparticles strongly induced apoptosis in human breast cancer cells with no significant impact on normal breast epithelial cells. In another study, the combination of venom and silica nanoparticles showed greater suppression of tumor growth in an in vivo model in nude mice compared to the venom alone [78].

In conclusion, this present review demonstrates that the venom from Malaysian common cobras could exert anticancer effects by modulating the cancer cell development mechanism and triggering apoptosis. The widespread availability of the cobras *N. kaouthia*, *N. sumatrana*, and *O. hannah* in the wild in Malaysia provides a valuable opportunity for researchers to further investigate their venom as a source of potential anticancer agents.

## Figures and Tables

**Table 1:** Comparison of the cobra species in Malaysia.

	*Naja kaouthia*	*Naja sumatrana*	*Ophiophagus hannah*
Common name	Monocled cobra	Equatorial spitting cobra	King cobra
Characteristics	Absence of occipitals, brown to greyish-brown body, with white circle hood mark	Absence of occipitals, black body, without hood mark, white marking on throat	Large head; small hood; adult has yellow, green, brown, or black body; presence of a pair of occipitals behind parietals
Length	Usually 4–5 feet, occasionally can reach up to 7.5 feet	Usually 3–3.9 feet, occasionally can reach up to 4.9 feet	Usually 8–18 feet
Distribution in Malaysia	Peninsular Malaysia, mainly in the northern part of peninsular Malaysia	Peninsular Malaysia, Sabah, and Sarawak	Peninsular Malaysia, Sabah, and Sarawak
Habitat	Not habitat-specific, can adapt to a wide range of habitats such as grassland and paddy fields	Not habitat-specific, can adapt to a wide range of habitats such as primary and secondary forests and human-surrounding environments	Habitat-specific, mainly inhabits forests
Proteomic composition of the venom	3FTx, PLA_2_, ohanin, CRVP, SVMP, vNGF, cardiotoxin, CVF, cytotoxin, and neurotoxin [36,37]	PLA_2_, neurotoxins, cardiotoxin, cytotoxin, 3FTX, CVF, SVMP, CRVP, natriuretic peptide, aminopeptidase, thaicobrin, complement-depleting factor, vNGF, and cobra serum albumin [38]	Natriuretic peptides, 3FTx, Kunitz-type inhibitor, PLA2, ohanin, CRVP, cystatin, insulin-like growth factor, SVMP, LAAO, SVSP, vNGF, vPDE, PLB, AChE, 5’NUC, and neprilysins [31,39]

Abbreviations: 3FTx—three-finger toxin, PLA_2_—phospholipase A_2_, CRVP—cysteine-rich venom protein, SVMP—snake venom metalloproteinase, vNGF—venom nerve-growth factor, CVF—cobra venom factor, LAAO—L-amino acid oxidase, vPDE—venom phosphodiesterase, SVSP—snake venom serine protease, PLB—phospholipase B, AChE—acetylcholinesterase, 5’NUC—5’-nucleotidase.

**Table 2:** Anticancer activity of Malaysian common cobra crude venom and protein components.

Species	Venom/Protein Component	Mechanism	Cancer Cell Type/Tissue	Reference
*Ophiophagus hannah*	Crude venom	Cytotoxic activity on pancreatic cancer cells (EC_50_; 1.39 ng/mL), reduced migration activity, and induction of apoptosis in PaTu 8988t cells	Patu 8988t	[45]
	Crude venom	Reduced tumor-cell-induced angiogenesis in vivo	Zebrafish embryos	[45]
	L-amino acid oxidase (OH-LAAO)	Antiproliferative activity on murine melanoma, human fibrosarcoma, and murine epithelial cells	B16/F10, HT1080, and Balb/3T3	[30]
	OH-LAAO	Cytotoxic activity on human breast adenocarcinoma cells (EC_50_ 0.05 µg/mL) and apoptosis induction	MCF-7	[33]
	OH-LAAO	Apoptosis induction and inhibition of prostate tumor growth	PC-3 xenograft in nude mice	[32]
	OH-LAAO	Induced alteration of gene expression involved in cytotoxicity and apoptotic effects	MCF-7	[31]
	OH-LAAO	Modulation of proteins involved in stress response, ubiquitination, proteolysis, cell proliferation, and apoptosis	MCF-7	[33]
*Naja kaouthia*	Crude venom	Cytotoxic activity on pancreatic cancer cells (EC_50_ 1.42 ng/mL)	PaTu 8988t	[45]
	Crude venom	Venom at a nonlethal dose inhibited tumor-cell proliferation and showed cytotoxic activity and apoptosis induction in human lung cancer cells and leukemic cells	Ehrlich-ascites cells (EAC), U937, K562	[47]
	Cytotoxin CT3	Histopathological changes in leukemia cells treated with CT3	A549 and HL60	[48]
	Cardiotoxic–cytotoxic protein	Antiproliferative activity and apoptosis induction in human leukemic cells	U947 and K562	[49]
	kaotree (*N. kouthia*) and atroporin (*Crotalus atrox*) combination	Elevated cytotoxic activity in various human cancer cells	HBL-100, BT-20, ZR-75-1, HT-29, and Diji	[50]
